# The Dose of Fungal Aerosol Inhaled by Workers in a Waste-Sorting Plant in Poland: A Case Study

**DOI:** 10.3390/ijerph17010177

**Published:** 2019-12-25

**Authors:** Ewa Brągoszewska

**Affiliations:** Department of Technologies and Installations for Waste Management, Faculty of Energy and Environmental Engineering, Silesian University of Technology, 18 Konarskiego St., 44 100 Gliwice, Poland; Ewa.Bragoszewska@polsl.pl; Tel.: +48-322-372-762

**Keywords:** indoor air quality, inhaled dose, bioaerosol, sorting plant, worker exposure, particle size distribution (PSD)

## Abstract

Bioaerosol monitoring is a rapidly emerging area in the context of work environments because microbial pollution is a key element of indoor air pollution and plays an important role in certain infectious diseases and allergies. However, as yet, relatively little is known about inhaled doses of microorganisms in workplaces. Today, the important issue of social concern is due to waste management, transport, sorting, and processing of wastes and their environmental impact and effects on public health. In fact, waste management activities can have numerous adverse effects on human wellbeing. Health effects are generally linked to exposure (EX), defined as the concentration of a contaminant and the length of time a person is exposed to this concentration. Dose is an effective tool for evaluating the quantity of a contaminant that actually crosses the body’s boundaries and influences the goal tissue. This document presents an analysis of the fungal waste-sorting plant EX dose *(FWSPED)* inhaled by workers in a waste-sorting plant (WSP) in Poland in March 2019. The main purpose of this research was to assess *FWSPED* inhaled by workers in two cabins at the WSP: the preliminary manual sorting cabin (PSP) and the purification manual sorting cabin (quality control; QSP). It was found that the *FWSPED* inhaled by workers was 193 CFU/kg in the PSP and 185 CFU/kg in the QSP. Fungal particles were quantitatively evaluated and qualitatively identified by the GEN III Biolog system. During the research, it was found that isolates belonging to the *Aspergilus flavus* and *Penicillum chrysogenum* strains were detected most frequently in the WSP. The total elimination of many anthropogenic sources is not possible, but the important findings of this research can be used to develop realistic management policies and methods to improve the biological air quality of WSPs for effective protection of WSP workers.

## 1. Introduction

Air contamination is responsible for three million early deaths globally each year, and it is the fifth top risk factor for mortality worldwide [1,2]. Indoor air is a complex and dynamic phenomenon in which physical factors and various type of pollutants can affect the health condition of occupants [3]. A wide range of health effects are associated with exposure (EX) to biological contaminants in work environments. Biological aerosols or bioaerosols include all particles with a biological source in suspension in the air (bacteria, microscopic fungi, viruses, pollen), as well as biomolecules (toxins and debris from membranes) [4].

Work in waste-sorting plants (WSPs) is associated with biological EX, which is considered an occupational health problem [5], and inhaling indoor air is the main means by which workers are exposed to biological agents. Biological aerosols represent a major risk potentially associated with acute and chronic adverse health effects and diseases, such as asthma, rhinitis, sinusitis, and bronchitis [6,7,8,9]. 

To date, concentrations of biological aerosols have been measured in many different environments [10,11,12,13,14], showing great variation in concentration values of air contaminants. However, the key factor responsible for the kinds of adverse health effects previously mentioned is not the total concentration but rather the dose of inhaled air pollutants. The dose is the amount of contaminant that actually crosses the body’s boundaries and reaches the inner body (for example, the respiratory tract epithelium) [15]. Concentration can, therefore, be used as a surrogate measurement of indoor EX, but it is effective only to the degree that it approximates concentrations actually experienced by every user in enclosed spaces [16]. 

When evaluating employee EX to bioaerosols, it is also necessary to identify the composition of species since their harmfulness to humans varies [4,17]. The air inside of WSPs is mainly characterized by the occurrence of fungi from the *Aspergillus* and *Penicillium* genera [18,19,20,21]. These contain species able to produce mycotoxins and pose a direct health risk to workers [17]. Moreover, microscopic fungi represent a highly diverse group, and different species may not have similar harmfulness [22]. In work environments where waste or compost is handled, spore counts of the *Penicillium* and *Aspergillus* genera are between two and four times higher than in domestic environments [23].

Since sorting workers are in direct contact with domestic hygiene waste, there is a risk of EX to microbiological contaminants that could pose a health risk [16]. Although EX to high levels of biological aerosols can be linked to deterioration of the human respiratory system, specific EX levels responsible for such effects are still unknown [24,25]. Occupational limits for EX to bioaerosols, proposed at local levels, include mainly mesophilic bacteria, endotoxins, and fungal spores [26]. The study of microbial indoor air quality in WSP air is not necessary in Poland, so such studies are rarely carried out, and the problem of microbiological hazards is often played down. The harmfulness of biological agents in Polish regulations is set out in the regulation on harmful biological factors for health in the work environment and protection of the health of employees exposed to these factors (document dated 22 April 2005) [27,28].

This study focuses on the following: (a) estimation of the inhaled dose of fungal aerosol in a WSP located in Poland; (b) identification of the dominant group of fungi in the WSP analyzed, and (c) determination of the particle size distribution (PSD) of fungal aerosol. The following paper aims to catalogue fungal aerosols in the WSP, thus increasing awareness of their possible health hazards. This may have wider implications for our knowledge of bioaerosols in an occupational setting.

## 2. Materials and Methods 

### 2.1. Sampling Site

The study was conducted in two cabins at a WSP for mixed municipal waste: preliminary manual sorting cabin (PSP) and purification manual sorting cabin (quality control; QSP), in Poland in March 2019. The samples of biological aerosols were collected at a height of approximately 1.2–1.5 m to simulate aspiration from the human inhalation zone. The elementary environmental parameters and a short description of the WSP are presented in Table 1.

**Figure 1:**
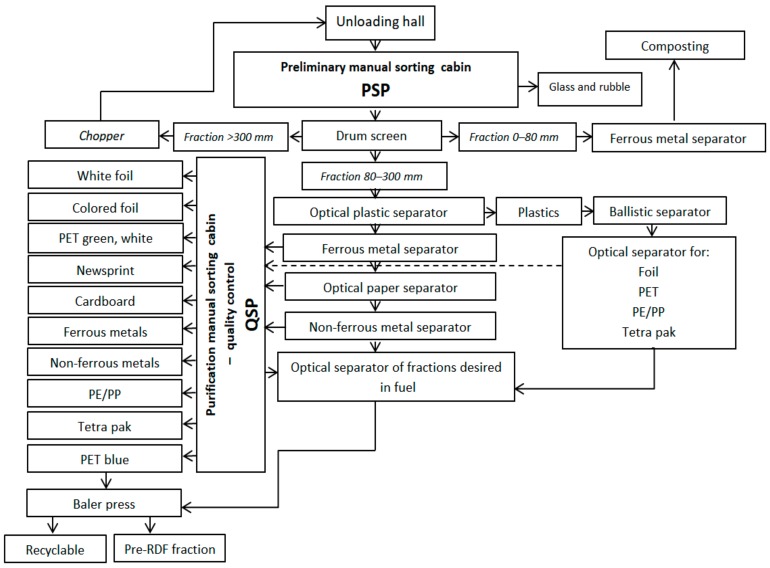
Scheme of the analyzed waste-sorting plant (WSP).

The sorting plant, which has a capacity of 70,000 Mg/year, works in a two-shift system and is equipped with technology adapted to segregate selectively collected municipal waste. During the research, about 20 Mg/h of mixed municipal waste was sorted (transported through PSP), about 30% of which went to the QSP and into which fractions were secreted by optopneumatic separators. The scheme of the analyzed WSP is shown in Figure 1.

### 2.2. Sampling and Analysis Methods 

Samples of fungal aerosol concentrations were taken using a six-stage Andersen cascade impactor (ACI) with cut-off diameters of 7.0, 4.7, 3.3, 2.1, 1.1, and 0.65 µm. The air flow was 28.3 dm^3^/min, and the sampling time was 10 min [29]. Before and after sampling, the flow rate was measured using a rotameter. ACI was disinfected using 70% ethanol-immersed cotton balls between every sampling. Malt extract agar (MEA 2%) was applied to the fungi, with chloramphenicol added to inhibit bacterial growth. The samples were incubated for five to six days at 26 °C. 

Total colony counts of fungal aerosol were revised for multiple impactions by the positive hole method and expressed as colony-forming units (CFUs) per cubic meter of air [30].Quality control was practiced in accordance with standards PN-EN12322 [31] and ISO 11133 [32], with the same operational details as in our earlier studies [33]. 

### 2.3. Identification of Selected Fungi 

A total of 433 isolates of fungi (229 in PSP, 204 in QSP) fungi were identified macro- and microscopically at the genus level according to the literature [34,35,36]. Based on these results, three main genera were selected for further biochemical analysis (*Aspergillus, Cladosporium,* and *Penicillum)*. 

Identification of fungal samples is still being carried out using traditional methods of macroscopic and microscopic analysis. The FF MicroPlate and database (Biolog, Hayward, CA, USA) provide an accurate method as an alternative to traditional methods.

The procedure involves five steps:

(1) Growing a pure fungus culture on a Petri dish with MEA 2% until enough conidiation is present to prepare a suspension;
(2) Swabbing the conidia on the surface of the agar plate and suspending to a specified density in FF inoculating fluid;
(3) Adding 100 μl of suspension into each well of the FF MicroPlate;
(4) Incubation of the FF MicroPlate at 26 °C for 24–96 hours;
(5) Reading the MicroPlates using the Biolog MicroStation™.

### 2.4. Calculation of the Fungal Waste-Sorting Plant Exposure Dose (FWSPED)

The EPA’s *Ex Factors Handbook* [37] and other publications [38,39] were the basis for the calculated *FWSPED*. Total concentrations of microscopic fungi were used to calculate the inhaled dose, and the calculations were based on the following equation:

(1) $$FWSPED=\;\frac{C\times IR\times IEF}{BW}$$

where *FWSPED* is the fungal WSP EX dose (CFU/kg); *C* is the level of fungal aerosol concentration (CFU/m³); *IEF* is the EX fraction (hours spent per day in the WSP) for the hourly diverse activity patterns (in sum, 8 hours); *IR* is the inhalation rate coefficient typical for particular activity intensities (m³/24hours) [37]; and *BW* is mean body weight (kg).

## 3. Results and Discussion

### 3.1. Fungal Waste-Sorting Plant Exposure Dose (FWSPED)

A worker’s time-budget survey was used to acquire information about workers’ activities during the day. A survey was used also to acquire information about staff weight. Table 2 presents a summary of the information obtained from this questionnaire, and Table 3 shows the results calculated for *FWSPED* and average concentration *(AVC)* of fungal aerosol. Short-term exposure values for inhalation are for males and females combined according to the age of the WSP staff [37].

In this analysis, a higher *FWSPED* was inhaled by workers in the PSP (193 CFU/kg). For example, the *FWSPED* was ten times higher than the fungal EX dose estimated to be inhaled by the staff of a public primary school in Egypt (18.2 CFU/kg) [40]. Previous research conducted in educational buildings in Poland has shown the fungal EX dose for staff to range from 17.8 to 73.3 CFU/kg [13]. 

The most significant environmental factor influencing the viability of microorganisms is temperature. Higher temperature could promote the growth of fungi [41,42]. This is confirmed by the higher concentration levels of fungal aerosol obtained in the PSP. 

The absorbed dose of air contaminants causes adverse health effects, and this could be one of the reasons the WSP workers suffer from various diseases [9,18,43]. It is, therefore, important that employees exposed to such aerosols use personal protective equipment (protection masks FFP3, footwear, protective clothing, and gloves), that buildings have an effective and efficient ventilation system, and that the time employees spend working in these conditions is limited.

### 3.2. Identification of Fungal Aerosol

The research analysis showed that all samples were polluted with at least one fungal species, and co-contamination with different fungi occurred in most of the samples. Following a comparison of the qualitative composition of the microorganisms isolated from air samples in the two cabins (PSP and QSP), a predominance of the following species was noted: in the PSP, the predominant species were *Aspergillus flavus* (31%), *Aspergillus phoenicis* (24.9%), *Penicillium chrysogenum* (26.6%), and *Cladosporium cladosporioides* (17.5%), while in the QSP, the predominant species were *Aspergillus flavus* (53.4%) and *Penicillium chrysogenum* (46.6%). These fungal species were also detected in air samples from waste-sorting facilities in several other studies [9,17,44,45].

The *Aspergillus* genus was first described by the Florentine priest and mycologist P. A. Micheli in 1729, and *Aspergillus flavus* species was described in 1809 by Link [46]. *Aspergillus flavus* may present high clinical relevance and should not be underestimated, since it constitutes a major risk to health in humans and animals [47]. *Aspergillus flavus* is the reason behind invasive allergic bronchopulmonary aspergillosis and is the most common cause of superficial infection [18,48,49]. 

The *Aspergillus spp.* produces aflatoxins that are carcinogenic to humans [50,51]. Daily inhalation of even low doses of aflatoxins causes chronic aflatoxicosis, resulting in immune suppression and possible liver cancer development [52,53]. High aflatoxin values produced by *Aspergillus flavus* were found in blood samples collected from employees of the WSP located in Portugal [54].

The second-most frequently identified *Aspergillus* genus in the WSP was *Aspergillus phoenicis.* This is an interesting heat-tolerant fungus that can synthesize enzymes and has several applications in the food industry due to its great hydrolytic potential [55]. This may explain its prevalence in the household waste of the analysed WSP.

The *Cladosporium* genus (first identified by Link in 1815) is most frequently found in spoiled organic matter in outdoor and indoor environments and is considered to be an important food contaminant [36,56,57]. *Cladosporium cladosporioides* is one of the most common species, distributed worldwide and present in many different decaying plants, food waste, air, and soil [58]. This fungus (widely distributed in the air) is reported to occasionally infect the lung, skin, eyes, and brain of humans, causing allergic mycoses [59]. 

In indoor air, a very common species of microscopic fungi is *Penicillum chrysogenum*, particularly in environments with high humidity, dampness or previous water damage, and it reproduces by producing spores which are known human allergens [59]. In general, *Penicillium* species have low pathogenicity, and infection is usually seen in immunocompromised individuals [60]. Sensitive persons may experience an adverse reaction, and they could suffer from breathing-related problems, skin allergies, and constant sneezing [61]. 

### 3.3. Particle Size Distribution (PSD) of Isolated Fungi

**Figure 2:**
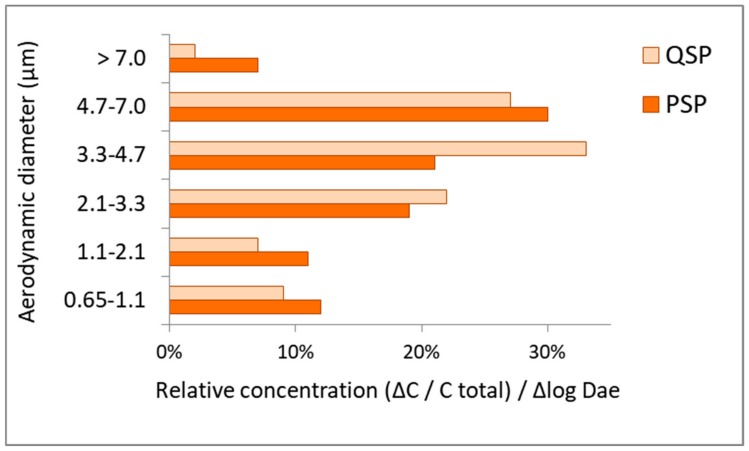
PSD of fungal aerosol in the analyzed WSP. D_ae_—aerodynamic diameter; ΔC—concentration of fungal aerosol on each stage of six-stage ACI. C_total_—total concentration of fungal aerosol; Δ log D_ae_—logarithm of differences of cut-off diameters for a particular stage of six-stage ACI.

The PSD of airborne fungi in the two WSP cabins (PSP and QSP) is shown in Figure 2.

Fungal spores usually range in size from 2 to 50 μm in diameter, with most allergenic spores in the respirable size range of 3 to 10 μm [62,63]. The PSD of fungal aerosol in the WSP was the highest for particles with an aerodynamic diameter of between 3.3 and 4.7 µm in the QSP, and between 4.7 and 7.0 µm in the PSP. Similar results were obtained in a WSP in Cracow, Poland, where the fungal PSD had a maximum peak ranging from 3.3 to 7.0 µm [64]. 

A large share of the fungal particles analyzed in the WSP was also observed in the 2.1–3.3 µm diameter range. Results obtained in this study correspond with the spore diameter of several of the species identified in the aerosol sample. For example, aerosols generated from pure *Penicillum chrysogenum* cultures had spores with a diameter between 2.8 and 4.0 μm [65]. 

## 4. Conclusions

The fungal waste-sorting plant exposure dose *(FWSPED)* inhaled by workers was 193 CFU/kg in the PSP and 185 CFU/kg in the QSP. This is ten times higher than the fungal EX dose inhaled by staff in a primary school in Poland [13].

During the research, it was found that isolates belonging to *Aspergilus flavus* and *Penicillum chrysogenum* strains were those most frequently detected in the WSP. Even low doses of aflatoxins produced by *Aspergillus flavus* can cause chronic aflatoxicosis when inhaled daily, resulting in immune suppression and possible development of liver cancer [52,53].

The particle size distributions (PSDs) of fungal aerosol in the WSP were the highest for particles with an aerodynamic diameter of between 3.3 and 4.7 µm in the QSP, and between 4.7 and 7.0 µm in the PSP. Fungal aerosol particles bigger than 3.3 µm comprised more than 50% of the total indoor concentration, increasing the health risk for exposed staff because most respirable allergenic spores range from 3 to 10 μm.

It is, therefore, important that employees exposed to such aerosols use personal protective equipment (protection masks FFP3, footwear, protective clothing, and gloves), that buildings have an effective and efficient ventilation system, and that the time employees spend working in these conditions is limited.

## Figures and Tables

**Table 1:** Environmental parameters and basic description of the two cabins: preliminary manual sorting cabin (PSP) and purification manual sorting cabin (quality control; QSP) at the waste-sorting plant (WSP).

	PSP	QSP
Ventilation system	Undergoes a 20-fold air exchange every hour (h); the cabins have supply and exhaust ventilation, and the air is drawn away from the conveyor belts.	
Volume (m^3^)	178	565
Number of occupants	6	10
Indoor temperature (°C)	18.3–21.4	17.1–19.4
Indoor relative humidity (%)	18–22	26–34
Outdoor temperature (°C) Outdoor relative humidity (%)	10.2–12.2 25.2–28	

**Table 2:** Description of the time budget and exposure (EX) dose for workers in the two WSP cabins (PSP and QSP).

Waste-Sorting Plant (WSP)		
Parameter	Short-Term Inhalation Rates by Activity Level/Minutes	
Activity Levels	Staff	Staff
	(m^3^/min)	(hour)
Sedentary/passive	0.0048	2
Light intensity	0.013	2
Moderate intensity	0.028	2
High intensity	0.052	2
Body weight (kg)	PSP 70; QSP 60	

**Table 3:** Calculated fungal waste-sorting plant exposure dose (*FWSPED*) inhaled by WSP staff and average concentration *(AVC)* of fungal aerosol ± SD.

	*FWSPED*—Waste-Sorting-Plant EX Dose (CFU/kg)	*AVC*—Average Concentration (CFU/m^3^)
PSP	193	8.1 ± 4.0 x 10^2^
QSP	185	7.2 ± 3.1 x 10^2^

*FWSPED*: Fungal Waste-Sorting Plant Exposure Dose; SD: standard deviation.
